# Lung failure after polytrauma with concomitant thoracic trauma in the elderly: an analysis from the TraumaRegister DGU®

**DOI:** 10.1186/s13017-022-00416-0

**Published:** 2022-02-23

**Authors:** Jan Tilmann Vollrath, Cora Rebecca Schindler, Ingo Marzi, Rolf Lefering, Philipp Störmann, the TraumaRegister DGU

**Affiliations:** 1 Department of Trauma, Hand, and Reconstructive Surgery, University Hospital Frankfurt am Main, Goethe University, Theodor-Stern-Kai 7, 60596 Frankfurt am Main, Germany; 2 Institute for Research in Operative Medicine (IFOM), Cologne Merheim Medical Center (CMMC), University of Witten/Herdecke, Cologne, Germany; 3 Committee on Emergency Medicine, Intensive Care and Trauma Management (Sektion NIS) of the German Trauma Society (DGU), Berlin, Germany

**Keywords:** Thoracic trauma, Geriatric patients, Lung failure

## Abstract

**Background:**

In developed countries worldwide, the number of older patients is increasing. Pulmonary complications are common in multiple injured patients with chest injuries. We assessed whether geriatric patients develop lung failure following multiple trauma with concomitant thoracic trauma more often than younger patients.

**Methods:**

A retrospective analysis of severely injured patients with concomitant blunt thoracic trauma registered in the TraumaRegister DGU® (TR-DGU) between 2009 and 2018 was performed. Patients were categorized into four age groups: 55–64 y, 65–74 y, 75–84 y, and ≥ 85 y. Adult patients aged 18–54 years served as a reference group. Lung failure was defined as PaO2/FIO2 ≤ 200 mm Hg, if mechanical ventilation was performed.

**Results:**

A total of 43,289 patients were included, of whom 9238 (21.3%) developed lung failure during their clinical stay. The rate of posttraumatic lung failure was seen to increase with age. While lung failure markedly increased the length of hospital stay, duration of mechanical ventilation, and length of ICU stay independent of the patient’s age, differences between younger and older patients with lung failure in regard to these parameters were clinically comparable. In addition, the development of respiratory failure showed a distinct increase in mortality with higher age, from 16.9% (18–54 y) to 67.2% (≥ 85 y).

**Conclusion:**

Development of lung failure in severely injured patients with thoracic trauma markedly increases hospital length of stay, length of ICU stay, and duration of mechanical ventilation in patients, regardless of age. The development of respiratory failure appears to be related to the severity of the chest trauma rather than to increasing patient age. However, the greatest effects of lung failure, particularly in terms of mortality, were observed in the oldest patients.

## Introduction

In the last decades, emergency physicians, especially in developed countries, have been faced with growing numbers of severely injured patients of advanced age [1]. In 2014, about 20,700 inpatient treatments per 100,000 inhabitants consisted of patients aged between 45 and 64, while 49,800 treatments per 100,000 inhabitants (more than twice as many) consisted of patients older than 65 years [2]. In Germany between 1990 and 2018, the number of people older than 67 years of age increased by 54% from 10.4 million to 15.9 million [3]. According to recent calculations by the Federal Statistical Office of Germany (Destatis), within the next 20 years, that number will grow to at least 20.9 million [3]. Parallel to this demographic development, a change in the mechanism of severe trauma can also be observed. In 1990, ground level falls accounted for 4.7% of major trauma cases, while by 2013, the same mechanism of injury accounted for 39.1% of major trauma cases [4, 5]. As ground level falls are a common mechanism of injury in the elderly, it can therefore be assumed that the number of severely injured patients in this age group increased in a similar way. In 2018, 27.1% of the patients included in the TraumaRegister DGU® were older than 70 years of age [6]. In this regard, several studies demonstrated increased mortality in elderly patients, and after adjusting for Injury Severity Score (ISS), these patients showed significantly longer intensive care unit (ICU) and in-hospital stays when compared to young patients suffering from multiple trauma [7–10].

Patients suffering from severe trauma are at risk of developing post-injury complications during their clinical course, including respiratory failure [11]. It is known that the breathing pattern changes with age, as resting elderly people have lower tidal volume and a higher respiratory rate and the ventilatory response to hypoxia or hypercapnia is significantly decreased [12]. Bearing this in mind, it is reasonable to assume that the occurrence of respiratory failure as a posttraumatic complication is more common in elderly patients and further worsens the outcome. Literature regarding trauma-induced lung failure in the elderly is scarce, especially with regard to higher incidence, greater severity of the course, and prognostic parameters. This TraumaRegister DGU® study was performed to investigate trauma-induced lung failure in the elderly after polytrauma with concomitant thoracic trauma.

## Methods

The TraumaRegister DGU® of the German Trauma Society (Deutsche Gesellschaft für Unfallchirurgie, DGU) was founded in 1993. The aim of this multi-centre database is a pseudonymized and standardized documentation of severely injured patients. Data are collected prospectively in four consecutive time phases from the site of the accident until discharge from hospital: (A) pre-hospital phase, (B) emergency room and initial surgery, (C) intensive care unit and (D) discharge. The documentation includes detailed information on demographics, injury pattern, comorbidities, pre- and in-hospital management, course on intensive care unit, relevant laboratory findings including data on transfusion and outcome of each individual. The inclusion criterion is admission to hospital via emergency room with subsequent ICU care or reach the hospital with vital signs and die before admission to ICU.

The infrastructure for documentation, data management, and data analysis is provided by the Academy for Trauma Surgery (AUC—Akademie der Unfallchirurgie GmbH), a company affiliated to the German Trauma Society. The scientific leadership is provided by the Committee on Emergency Medicine, Intensive Care and Trauma Management (Sektion NIS) of the German Trauma Society. The participating hospitals submit their data pseudonymised into a central database via a web-based application.

The participating hospitals are primarily located in Germany (90%), but a rising number of hospitals of other countries contribute data as well (at the moment from Austria, Belgium,, Finland, Luxembourg, Slovenia, Switzerland, The Netherlands, and the United Arab Emirates). Currently, approx. 30,000 cases from more than 650 hospitals are entered into the database per year.

Participation in TraumaRegister DGU® is voluntary. For hospitals associated with TraumaNetzwerk DGU®, however, the entry of at least a basic data set is obligatory for reasons of quality assurance. Scientific data analysis is approved according to a peer review procedure established by Sektion NIS. The present study is in line with the publication guidelines of the TraumaRegister DGU® (TR-DGU) and registered as TR-DGU project ID 2019–056.

This retrospective study included patients from the standard data set of the TR-DGU between 2009 and 2018 suffering from a blunt thoracic trauma. There should be at least one serious injury (Maximum Abbreviated Injury Scale (MAIS) score ≥ 3) to any body region. This was not necessarily located in the thorax. Due to the similarity of the health care systems, only patients from Germany, Austria, and Switzerland were included. Patients were excluded based on the following criteria: age < 18 years; penetrating thoracic injuries, as well as missing documentation of whether the thoracic injury was blunt or penetrating; early transfer to another hospital; no ICU stay; and missing documentation of organ failure. The Injury Severity Score (ISS) and injuries related to different body regions were determined using the Abbreviated Injury Scale (AIS, version 2005, update 2008 [13]. For further analysis, elderly patients were categorized into four age groups: 55–64 y, 65–74 y, 75–84 y, and ≥ 85 y. Adult patients aged 18–54 years served as a reference group. To analyze the influence of a hemo-/pneumothorax on the development of lung failure in these age groups, patients with hemo-/pneumothorax with or without lung failure were further categorized according to the AIS severity. The same analysis was performed for rib fractures. Lung failure was documented during intensive care treatment and defined as PaO2/FIO2 ≤ 200 mm Hg, if mechanical ventilation was performed, which is equivalent to 3 or 4 points for the respiratory system in the Sequential Organ Failure Assessment (SOFA) score. This criterion must be met for at least two days for the condition to be considered lung failure.

### Statistics

Data are presented as mean ± standard deviation (SD) for continuous variables and percentages (%) for categorical variables. In case of considerable skewness, median with inter-quartile range (IQR) was used. However, the robustness of the median sometimes hides group differences, and therefore the mean value was used in addition for graphical comparisons. Formal statistical testing comparing cohorts was avoided since, due to the large sample size, even minor, clinically not relevant differences would result in highly statistically significant results, which might lead to an overinterpretation. Logistic regression analysis was used to evaluate the effect of age, injury severity and type of injury on lung failure (dependent variable) in patients with mechanical ventilation on ICU. Statistical analysis was performed using Statistical Package for Social Sciences (SPSS, version 25, IBM Corp., Armonk, New York, United States). The figures were created using Microsoft Excel (version 16.48, Microsoft Cooperation, Redmond, Washington, United States).

## Results

**Fig. 1:**
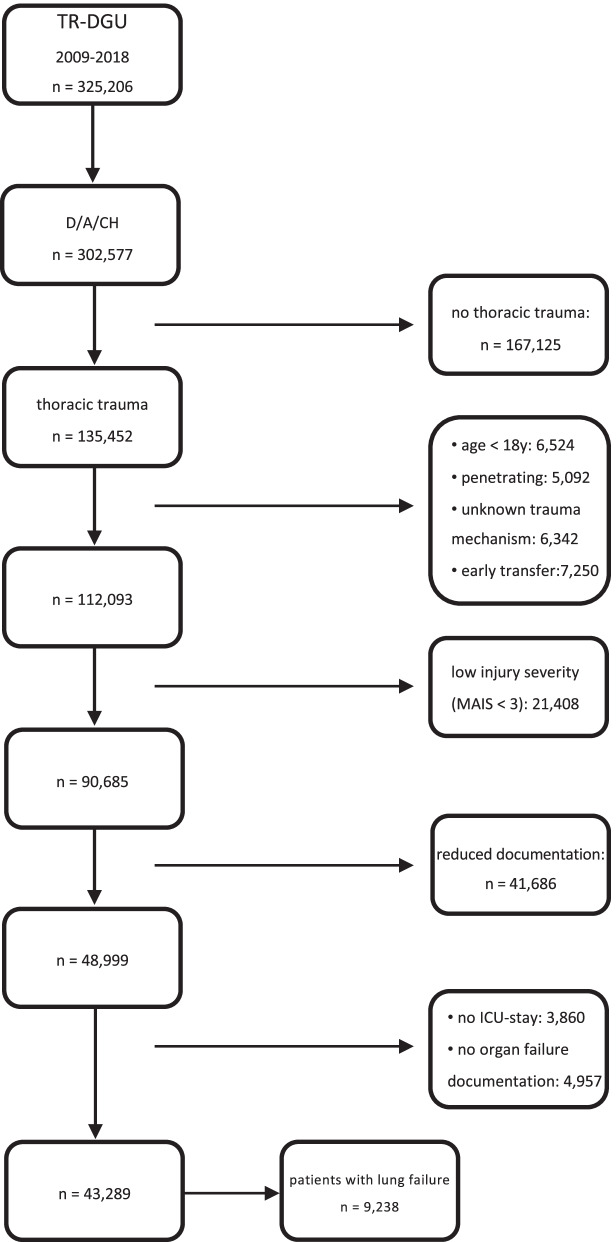
Flowchart study population. *Abbreviations* ICU, Intensive Care Unit; MAIS, Maximum Abbreviated Injury Scale; D, Germany; A, Austria; CH, Switzerland

In total, 135,452 patients with thoracic trauma were documented between 2009 and 2018 in German, Austrian, and Swiss hospitals. 92,163 patients were excluded according to the exclusion criteria depicted in detail in Fig. 1. Finally, 43,289 patients were included in further analyses, of whom 9238 (21.3%) developed lung failure during their clinical course (Fig. 1).

Basic patient data are shown in Table 1. 24,160 patients (55.8%) were in the adult reference group, while the number of elderly patients ranged from 1671 (age 85+) to 7113 (Table 1). The proportion of male patients decreased in the older age groups, so that in the oldest group, sex distribution was nearly balanced. The overall ISS was 26.1 ± 12.4, and it was comparable between all age groups (Table 1).

**Table 1:** Demographic characteristics of the study population

Patient characteristics	Total	18–54 y	55–64 y	65–74 y	75–84 y	≥ 85 y
Total (*n*, %)	43,289 (100%)	24,160 (55.8%)	7113 (16.4%)	5462 (12.6%)	4883 (11.3%)	1671 (3.9%)
Sex (male, %)	32,025 (74.2%)	18,695 (77.6%)	5448 (76.7%)	3932 (72.2%)	3115 (63.9%)	835 (50.1%)
Trauma mechanism (*n*, %)						
Car collision	12,484 (29.0%)	8251 (34.3%)	1629 (23.0%)	1181 (21.7%)	1157 (23.8%)	266 (16.0%)
Motorcycle collision	6681 (15.5%)	4856 (20.2%)	1207 (17.0%)	441 (8.1%)	168 (3.5%)	9 (0.5%)
Bicycle collision	3437 (8.0%)	1490 (6.2%)	716 (10.1%)	671 (12.3%)	483 (9.9%)	77 (4.6%)
Pedestrian	2769 (6.4%)	1210 (5.0%)	417 (5.9%)	449 (8.3%)	511 (10.5%)	182 (11.0%)
Fall > 3 m	8636 (20.0%)	4888 (20.3%)	1551 (21.9%)	1196 (22.0%)	798 (16.4%)	203 (12.2%)
Fall < 3 m	5921 (13.7%)	1405 (5.8%)	996 (14.1%)	1168 (21.5%)	1512 (31.1%)	840 (50.5%)
Others	3182 (7.4%)	1965 (8.2%)	568 (8.0%)	330 (6.1%)	234 (4.8%)	85 (5.1%)
ISS ± SD (points)	26.1 ± 12.4	26.4 ± 12.5	25.6 ± 12.0	26.0 ± 12.2	26.0 ± 12.4	25.9 ± 12.5
ISS ≥ 16 (*n*, %)	35,342 (81.6%)	19,835 (82.1%)	5676 (79.8%)	4477 (82.0%)	3994 (81.8%)	1360 (81.4%)

ISS, Injury Severity Score; SD, standard deviation

All patients had a thoracic trauma, and this was severe (AIS ≥ 3) in 36,428 cases (84.1%); this relation was similar in all age subgroups. The proportion of patients suffering from a severe head injury (AIS_Head_ ≥ 3) increased with age (Table 2).

**Table 2:** Specific injury pattern

	Total	18–54 y	55–64 y	65–74 y	75–84 y	≥ 85 y
AIS head ≥ 3 points (*n*, %)	16,226 (37.5%)	8632 (35.7%)	2402 (33.8%)	2276 (41.7%)	2132 (43.7%)	784 (46.9%)
AIS thorax ≥ 3 (*n*, %)	36,428 (84.1%)	19,940 (82.5%)	6191 (87.0%)	4698 (86.0%)	4202 (86.1%)	1393 (83.4%)
AIS abdomen ≥ 3 (*n*, %)	6835 (15.8%)	4628 (19.2%)	952 (13.4%)	671 (12.3%)	465 (9.5%)	119 (7.1%)
AIS extremity ≥ 3 (*n*, %)	13,409 (31.0%)	8551 (35.4%)	1971 (27.7%)	1351 (24.7%)	1154 (23.6%)	382 (31.0%)
Rib fractures						
None	10,542 (24.4%)	7757 (32.1%)	1068 (15.0%)	772 (14.1%)	682 (14.0%)	263 (15.7%)
AIS 1	2604 (6.0%)	1575 (6.5%)	364 (5.1%)	289 (5.3%)	273 (5.6%)	103 (6.2%)
AIS 2	4320 (10.0%)	2514 (10.4%)	679 (9.5%)	528 (9.7%)	453 (9.3%)	146 (8.7%)
AIS 3	19,961 (46.1%)	9694 (40.1%)	3806 (53.5%)	2911 (53.3%)	2639 (54.0%)	911 (54.5%)
AIS 4	3404 (7.9%)	1556 (6.4%)	714 (10.0%)	548 (10.0%)	449 (9.2%)	137 (8.2%)
AIS 5	2458 (5.7%)	1064 (4.4%)	482 (6.8%)	414 (7.6%)	387 (7.9%)	111 (6.6%)
Hemothorax (*n*, %)	8157 (18.8%)	4289 (17.8%)	1383 (19.4%)	1122 (20.5%)	1032 (21.1%)	331 (19.8%)
Pneumothorax (*n*, %)	16,704 (38.6%)	10,078 (41.7%)	2721 (38.3%)	1891 (34.6%)	1567 (32.1%)	447 (26.8%)
Lung contusion (*n*, %)	20,461 (47.3%)	13,447 (55.7%)	2927 (41.2%)	2002 (36.7%)	1642 (33.6%)	443 (26.5%)
Lung laceration (*n*, %)	997 (2.3%)	689 (2.9%)	125 (1.8%)	92 (1.7%)	71 (1.5%)	20 (1.2%)

AIS, abbreviated Injury Scale

Pneumothorax and lung contusion/laceration were more prevalent in younger patients, while rib fractures were more common in patients older than 55 years of age (Table 2). An overview of the specific injury pattern is depicted in Table 2.

While the number of patients who were mechanically ventilated during their ICU stay did not increase with age, in-hospital mortality was higher in the older patients (Table 3). Furthermore, the number of patients developing lung failure during the clinical course was higher for patients older than 65 years of age when compared to both groups of younger patients (18–54 y: 19.4%; 55–64 y: 20.5%; 65–74 y: 24.7%; 75–84 y: 26.8%; ≥ 85 y: 25.9%; Table 3). Additionally, the number of patients with multi-organ failure and sepsis also increased with age (Table 3). Patients of older age underwent surgery less frequently, and chest tubes were used less often in those patients. The proportion of patients who received a blood transfusion was comparable across all age groups (Table 3).

**Table 3:** Interventions and outcome

	Total	18–54 y	55–64 y	65–74 y	75–84 y	≥ 85 y
Lung failure (*n*, %)	9238 (21.3%)	4688 (19.4%)	1461 (20.5%)	1348 (24.7%)	1308 (26.8%)	433 (25.9%)
Length of stay on ICU (days), median [IQR]	5 [2–14]	5 [2–14]	5 [2–14]	6 [2–16]	6 [2–15]	4 [2–10]
Mechanical ventilation on ICU (*n*, %)	25,014 (57.8%)	14,327 (59.3%)	3782 (53.2%)	3095 (56.7%)	2861 (58.6%)	949 (56.8%)
If ventilated, duration of mechanical ventilation (days) median [IQR]	5 [1–14]	4 [1–13]	6 [1–16]	6 [2–17]	5 [1–15]	3 [1–8]
Chest tube (*n*, %)	2142 (5.7%)	1303 (6.2%)	370 (6.1%)	235 (5.1%)	190 (4.7%)	44 (3.1%)
Operation (*n*, %)	31,784 (73.4%)	18,625 (77.1%)	5216 (73.3%)	3819 (69.9%)	3147 (64.4%)	977 (58.5%)
Blood transfusion in the ER (*n*, %)	6445 (14.9%)	3839 (15.9%)	938 (13.2%)	764 (14.0%)	659 (13.5%)	245 (14.7%)
Sepsis (*n*, %)	3453 (8.2%)	1620 (6.9%)	632 (9.1%)	527 (10.0%)	520 (11.1%)	154 (9.6%)
Multiple organ failure (*n*, %)	11,895 (27.5%)	6045 (25.0%)	1892 (26.6%)	1663 (30.4%)	1668 (34.6%)	607 (36.3%)
In-hospital mortality (*n*, %)	4910 (11.3%)	1621 (6.7%)	634 (8.9%)	785 (14.4%)	1166 (23.9%)	704 (42.1%)
Length of hospital stay (days), median [IQR]	16 [9–27]	16 [9–27]	16 [9–28]	17 [9–28]	15 [8–26]	11 [4–20]
Survivor only: Length of hospital stay (days), median [IQR]	17 [10–28]	17 [10–28]	17 [10–29]	18 [11–29]	18 [11–29]	15 [19–24]

ER, emergency room; ICU, intensive care unit

**Fig. 2:**
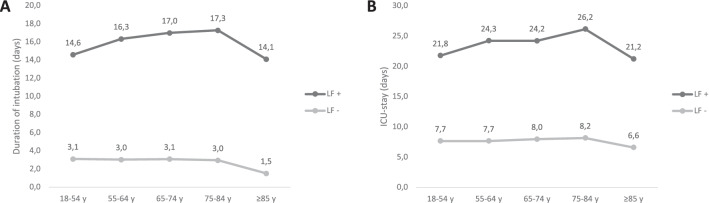
Duration of intubation and ICU stay. The duration of intubation (**A**) and ICU stay (**B**) of all patients with or without lung failure are shown for the different age groups. *Abbreviations* LF−, without lung failure; LF+, with lung failure; y, years

Without the development of lung failure, the duration of mechanical ventilation did not differ between the age groups except for patients older than 85 years of age, whose ventilation time was only half as long (Fig. 2A).

Independent of age, lung failure markedly extended the duration of mechanical ventilation. Again, in patients older than 65 years of age, the longest mean times for mechanical ventilation were registered, except for the oldest group, which had shorter ventilation times (Fig. 2A).

Patients who did not suffer from respiratory failure had a comparable duration of their ICU treatment, while the development of lung failure clearly increased the length of ICU stays. For both, the duration of ICU treatment was longer for the older groups, except for patients who were older than 85 years of age.

**Fig. 3:**
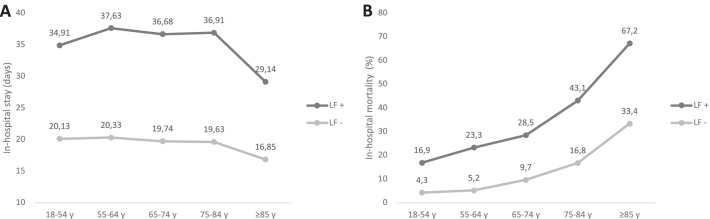
Duration of in-hospital stay and in-hospital mortality. The duration of in-hospital stay (**A**) and in-hospital mortality (**B**) of all patients with or without lung failure are shown for the different age groups. *Abbreviations* LF−, without lung failure; LF+, with lung failure; y, years

The mean hospital length of stay was comparable between the age groups but was clearly prolonged if the patients developed lung failure (18–54 y: 20.1 d vs. 34.9 d; 55–64 y: 20.3 d vs. 37.6 d; 65–74 y: 19.7 d vs. 36.7 d; 75–84 y: 19.4 d vs. 36.9 d; ≥ 85 y: 16.9 d vs. 29.1 d; Fig. 3A).

In-hospital mortality in patients who did not suffer from respiratory failure increased with age (Fig. 3B). If patients developed lung failure, mortality distinctly increased throughout all age groups, with the highest rates for the elderly (18–54 y: 16.9%; 55–64 y: 23.3%; 65–74 y: 28.5%; 75–84 y: 43.1%; ≥ 85 y: 67.2%; Fig. 3B).

**Fig. 4:**
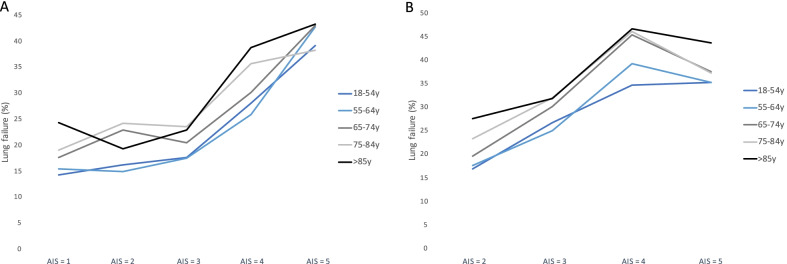
Rate of lung failure according to the Abbreviated Injury Scale. The rate of lung failure (%) according to the AIS severity for rib fractures (**A**) or hemo-/pneumothorax (**B**) is shown for the different age groups. AIS rib fracture: AIS 1: Fracture, 1 rib; AIS 2: Fracture, 2 ribs; AIS 3: Fracture, ≥ 3 ribs or multi-fragment fractures of 3–5 ribs (unstable thorax), unilateral; AIS 4: Multi-fragment fractures of > 5 ribs (unstable thorax), unilateral; AIS 5: Multi-fragment fractures (unstable thorax), bilateral. *Abbreviations* AIS, Abbreviated Injury Scale

To assess the influence of the severity of rib fractures and of hemo-/pneumothorax on the development of respiratory failure, we analyzed the rate of failure in the different age groups in regard to the AIS for both injury patterns (Fig. 4A, B). For rib fractures, in cases of injury severity of AIS ≥ 3, patients suffered from respiratory failure more often (Fig. 4A). While the rate of lung failure was higher for older patients, the increase between the age groups was comparable. Commensurable results were found for the different peculiarities of hemo-/pneumothorax as shown in Fig. 4B.

After adjustment for the different age groups and individual thoracic injuries (hemothorax, pneumothorax, lung contusion, lung laceration or rib fractures ≥ 3) in a logistic regression model, all covariables, except pneumothorax (*p* < 0.1), were found to exert a significant effect on the development of lung failure (Table 4). The overall mild effects are slightly higher for the covariable age than for the individual thoracic injuries (Table 4).

**Table 4:** Multivariable regression analysis for development of lung failure in patients with mechanical ventilation on ICU (*n* = 25,014)

	Coefficent	*p* value	OR	95% CI of OR
ISS (per point)	0.020	< 0.001	1.020	1.018–1.022
AIS severity of thorax trauma (per point)	0.161	< 0.001	1.18	1.13–1.22
Pre-injury ASA 3/4	0.396	< 0.001	1.49	1.37–1.61
Age group (reference: 18–54 y)		< 0.001		
55–64 y	0.241	< 0.001	1.27	1.18–1.38
65–74 y	0.414	< 0.001	1.51	1.39–1.65
75–84 y	0.409	< 0.001	1.51	1.37–1.66
≥ 85 y	0.412	< 0.001	1.51	1.31–1.75
Hemothorax	0.352	< 0.001	1.42	1.33–1.52
Pneumothorax	0.000	0.99	1.00	0.94–1.06
Lung contusion	0.222	< 0.001	1.25	1.18–1.32
Lung lacteration	0.142	0.07	1.15	0.99–1.34
Rib fractures (3 or more)	0.054	0.09	1.06	0.99–1.12
Constant	− 2.153	< 0.001		

ASA, American Society of Anesthesiologists physical status classification system; CI, confidence interval; ISS, Injury Severity Score; OR, odds ratio, y years

## Discussion

### Mortality

The increasing number of severely injured elderly is challenging due to numerous age-related factors. In this respect, increased morbidity and mortality have been reported in several prior studies [7–10]. In our recent study, we observed higher mortality and an increasing number of patients of advanced age suffering from multi-organ failure, thus confirming observations from previous studies.

As expected, we found a higher rate of lung failure in patients older than 65 years of age in our collective. Nearly independent of age, development of pulmonary failure clearly increased length of ICU stay, length of hospital stay, and mortality. Nevertheless, patients in the oldest age group (≥ 85 y) showed shorter duration of mechanical ventilation, ICU treatment, and in-hospital stay when compared to the younger age groups. This might be due to this group presenting the highest mortality rate and early relocation to rehabilitation facilities, resulting in an overall shorter treatment [14]. In line, shorter ICU stays for patients ≥ 80 years have also been observed in other studies [15]. Wutzler et al. investigated the probability of lung failure in multiple injured patients with thoracic trauma and identified seven independent predictors with a significant correlation with respiratory failure [16]. While the highest odds ratios (ORs) were observed in cases of AIS_Thorax_ = 5 points (1.58), surgical intervention (1.71), and multiple surgeries (2.41), the lowest OR was observed for age (1.02) (13), which is in line with our results. Navarrete-Navarro et al. investigated factors related to ICU mortality from trauma-related acute respiratory distress syndrome (ARDS) and reported an age-related increase [17]. Although these data are not completely comparable to our study due to different age groups and slightly different trauma mechanisms, the results confirm an age-related increase in hospital mortality in trauma-related lung failure. Age-related mortality following blunt thoracic trauma has also been observed by Huber et al. [18]. Furthermore, an age of 60 years or older has been shown to be an independent risk factor for the development of pulmonary failure after severe trauma, which is in line with the results of our recent study [19]. In a multicenter, prospective cohort study of patients with severe respiratory failure, older age was shown to be associated with increased hospital mortality (OR 1.03) [20]. Moreover, incidence of acute lung injury and mortality increased with age [21]. This is in line with our results showing an in-hospital mortality of 67.5% when patients older than 85 years of age develop lung failure. Nevertheless, it must be mentioned that the two studies cited investigated lung failure with pulmonary and extrapulmonary etiology, while our study focuses on polytrauma with concomitant thoracic trauma.

### Length of mechanical ventilation and ICU stay

With regard to the length of mechanical ventilation and the length of ICU and hospital stay in patients without lung failure, we did not observe clear age-dependent tendencies. The occurrence of respiratory failure resulted in a pronounced prolongation of these lengths, but interestingly, patients from the younger groups were affected similarly to the elderly. This is in line with a study by Santana-Cabrera et al. showing no influence of age on the duration of ICU stay in patients treated in the ICU for ≥ 14 days [22]. On the other hand, age has been shown to be a parameter that prolongs ICU stay in trauma patients [15]. However, in line with our study, the number of additional days spent in the ICU due to patients’ age was rather low compared to other factors [15]. Lung failure has been shown to be among the factors that most influenced the prolongation of ICU stay in trauma patients [15]. This is in line with our study, showing significantly longer length of ICU and in-hospital stay in patients developing lung failure. No differences in the length of ICU stay have been reported for advanced-age motorcycle trauma, but among mechanically ventilated patients, the older group had more ventilator days [23]. With regard to the duration of ventilation, we saw no difference between the age groups when no respiratory failure occurred, whereas the occurrence of respiratory failure tended to increase the duration of ventilation somewhat in the older patients. Due to changes in respiratory mechanics and the reduced ability of the elderly to compensate, we expected a more distinct increase in the duration of ventilation. An age of 65 years or older has been shown to be a predictor of prolonged mechanical ventilation after cardiac surgery (OR 1.296) [24]. To date, studies investigating predictors of prolonged ventilatory support in the trauma population are scarce. Nevertheless, age has been demonstrated to be a predictor of the need for prolonged ventilatory support, which is likely a reflection of patients’ comorbidities [25, 26]. Dimopoulou et al. [27] revealed that in thoracic trauma patients admitted to the ICU, prolonged mechanical ventilation was primarily determined by the presence of bilateral chest injuries, age, and the degree of concomitant neurotrauma. Features of neurologic impairment, such as poor consciousness and poor ability to clear secretions, have been shown to be the most common causes of respiratory failure in patients with prolonged mechanical ventilation [28]. A longer duration of ventilation in the elderly could therefore also be partly explained by the more frequent occurrence of neurotrauma in this age group.

### Rib fractures and hemo-/pneumothorax

Rib fractures are a common thoracic injury, and elderly patients are especially prone to fracturing ribs due to loss of cortical bone mass and stiffening of the thoracic cage [29]. For the development of respiratory failure in the presence of rib fractures, we expected higher rates in the elderly due to lower compensatory capacity. Contrary to our assumption, the increase in the rate of respiratory failure as a function of injury severity for this entity was comparable for patients ≥ 65 years of age having only slightly higher rates of lung failure compared to younger patients. In the elderly patients, the increase of respiratory failure at the highest injury severity was lower, which was again associated with the tremendously increased mortality in this age group. In a retrospective cohort study, Bulger et al. revealed that patients ≥ 65 years of age with rib fractures after blunt chest trauma have twice the mortality and thoracic morbidity of younger patients with similar injuries [30]. The authors showed that with each additional rib fracture in the elderly, mortality increases by 19% and risk of pneumonia by 27% [30]. Furthermore, ARDS rates increased as the number of rib fractures increased, with overall higher rates in the elderly [30]. In contrast, Holcomb et al. reported that patients older than 45 years of age who have more than four ribs fractured are already at risk of prolonged ICU stays, ventilator days, and overall hospital days [31]. Furthermore, these patients showed higher rates of pulmonary complications (including ARDS), although this difference was not statistically significant [31]. The number of rib fractures has been shown to correlate directly with an increasing rate of lung failure and mortality [29]. However, the association between age and the number of rib fractures was only slight [29], which is comparable to the results presented. Several factors, including age ≥ 65 years, total number of fractured ribs, and presence of bilateral fractures, have been shown to contribute to the morbidity and mortality of chest wall injuries [32]. This is in line with our study showing higher rates of lung failure for patients ≥ 65 years, for patients with multifragmentary fractures of more than five ribs, and for bilateral multifragmentary rib fractures. Pape et al. observed that the association between rib fractures and chest-related death was low unless bilateral injuries have been detected [33]. Furthermore, injuries to the lung parenchyma are associated with chest-related death, especially if the injuries are bilateral or associated with hemopneumothorax [33]. With regard to the extent of hemo-/pneumothoraces, we have also observed comparable incidence of lung failure between the age groups. The tendency for higher rates of respiratory failure in the older groups may also be explained in part by the higher proportion of neurotrauma in these age groups, as mentioned before. However, literature dealing with the association between pneumo-/hematothoraces and the occurrence of respiratory failure is scarce. In line with our study, Schulz-Drost et al. observed a higher incidence of lung contusion in young patients than in the elderly after polytrauma with concomitant thoracic trauma [14]. On the other hand, the authors observed increasing incidence of flail chest with growing age in the group of severe lung contusion and that older patients are less likely to be discharged home [14].

### Limitations

The major limitation of this study arises from its methodology, as the study is strictly retrospective. Moreover, in the TraumaRegister DGU®, diagnoses are only identified by their AIS codes; thus, no absolutely strict description of each individual patient’s injuries is given. Whether the lung failure is a consequence of the multiple injury or the thoracic injury cannot be assessed with absolute certainty on the basis of this trauma registry evaluation. Furthermore, health and medical histories, as well as frailty, which are particularly relevant in geriatric patients, are not recorded in the database. Neither the exact cause of lung failure nor the exact time of occurrence is documented in the TraumaRegister DGU®. The influence on premature limitation of treatment when an advance directive is available cannot be inferred from the registry data. Furthermore, the indication for certain measures, such as the placement of a chest tube in the presence of a pneumothorax, cannot be verified in the trauma registry.

## Conclusions

The development of lung failure after blunt thoracic trauma in multiple injured patients leads to a significant increase in the duration of mechanical ventilation and intensive care stays. This phenomenon seems to be only slightly influenced by higher age. If patients develop respiratory failure, mortality increases markedly, which is most pronounced in the elderly. The occurrence of respiratory failure increases depending on the extent of rib fractures or hemato/pneumothoraces but appears to affect the different age groups similarly. Therefore, after blunt thoracic trauma in multiple injured patients, lung protective treatment and close monitoring should be established regardless of patient age to avoid the development of lung failure.

## Data Availability

None.

## Abbreviations

A: Austria
AIS: Abbreviated Injury Scale
ARDS: Acute Respiratory Distress Syndrome
ASA: American Society of Anesthesiologists
AUC: Akademie der Unfallchirurgie GmbH/Academy for Trauma Surgery
CH: Switzerland
CI: Confidence interval
CMMC: Cologne Merheim Medical Center
D: Germany
Destatis: Federal Statistical Office of Germany
DGU: Deutsche Gesellschaft für Unfallchirurgie/ German Trauma Society
ER: Emergency room
ICU: Intensive care unit
IFOM: Institute for Research in Operative Medicine
IMC: Intermediate care unit
ISS: Injury Severity Score
IQR: Inter-quartile range
LF: Lung failure
MAIS: Maximum Abbreviated Injury Scale
OD: Odds ratio
SD: Standard deviation
SOFA: Sequential Organ Failure Assessment Score
SPSS: Statistical Package for Social Sciences
TR-DGU: TraumaRegister DGU®
Sektion NIS: Committee on Emergency Medicine, Intensive Care and Trauma Management of the German Trauma Society
Y: Years

## References

[1] Joseph A (2015). Trauma in the elderly: burden or opportunity?. Injury.

[2] Federal Statistical Office (Destatis). Older people in Germany and the EU. 2016.

[3] Federal Statistical Office (Destatis). A changing population: assumptions and results of the 14th coordinated population projection. 2019.

[4] Hashmi A, Ibrahim-Zada I, Rhee P, Aziz H, Fain MJ, Friese RS (2014). Predictors of mortality in geriatric trauma patients: a systematic review and meta-analysis. J Trauma Acute Care Surg.

[5] Fisher JM, Bates C, Banerjee J (2017). The growing challenge of major trauma in older people: a role for comprehensive geriatric assessment?. Age Ageing.

[6] Sektion NIS der DGU/AUC. Jahresbericht 2019. 2019.

[7] Carpenter CR, Arendts G, Hullick C, Nagaraj G, Cooper Z, Burkett E (2017). Major trauma in the older patient: evolving trauma care beyond management of bumps and bruises. Emerg Med Australas.

[8] Earl-Royal E, Kaufman EJ, Hsu JY, Wiebe DJ, Reilly PM, Holena DN (2016). Age and preexisting conditions as risk factors for severe adverse events and failure to rescue after injury. J Surg Res.

[9] Mörs K, Wagner N, Sturm R, Störmann P, Vollrath JT, Marzi I (2019). Enhanced pro-inflammatory response and higher mortality rates in geriatric trauma patients. Eur J Trauma Emerg Surg..

[10] Fu C-Y, Bajani F, Bokhari M, Starr F, Messer T, Kaminsky M, et al. Age itself or age-associated comorbidities? A nationwide analysis of outcomes of geriatric trauma. Eur J Trauma Emerg Surg. 2021;1–8. 10.1007/s00068-020-01595-8 PMC7839290 33502566

[11] Pfeifer R, Tarkin IS, Rocos B, Pape H-C (2009). Patterns of mortality and causes of death in polytrauma patients—has anything changed?. Injury.

[12] Sprung J, Gajic O, Warner DO (2006). Review article: age related alterations in respiratory function—anesthetic considerations. Can J Anaesth.

[13] www.aaam.org.

[14] Schulz-Drost S, Finkbeiner R, Lefering R, Grosso M, Krinner S, Langenbach A (2019). Lung contusion in polytrauma: an analysis of the TraumaRegister DGU. Thorac Cardiovasc Surg..

[15] Böhmer AB, Just KS, Lefering R, Paffrath T, Bouillon B, Joppich R (2014). Factors influencing lengths of stay in the intensive care unit for surviving trauma patients: a retrospective analysis of 30,157 cases. Crit Care BioMed Cent.

[16] Wutzler S, Wafaisade A, Maegele M, Laurer H, Geiger EV, Walcher F (2012). Lung Organ Failure Score (LOFS): probability of severe pulmonary organ failure after multiple injuries including chest trauma. Injury.

[17] Navarrete-Navarro P, Rodriguez A, Reynolds N, West R, Rivera R, Scalea T (2001). Adult respiratory distress syndrome among blunt and penetrating trauma patients: demographics, mortality, and resource utilization over 8 years. J Crit Care.

[18] Huber S, Biberthaler P, Delhey P, Trentzsch H, Winter H, van Griensven M (2014). Predictors of poor outcomes after significant chest trauma in multiply injured patients: a retrospective analysis from the German Trauma Registry (Trauma Register DGU®). Scand J Trauma Resusc Emerg Med.

[19] Geiger EV, Lustenberger T, Wutzler S, Lefering R, Lehnert M, Walcher F (2013). Predictors of pulmonary failure following severe trauma: a trauma registry-based analysis. Scand J Trauma Resusc Emerg Med..

[20] Laffey JG, Bellani G, Pham T, Fan E, Madotto F, Bajwa EK (2016). Potentially modifiable factors contributing to outcome from acute respiratory distress syndrome: the LUNG SAFE study. Intensive Care Med..

[21] Rubenfeld GD, Caldwell E, Peabody E, Weaver J, Martin DP, Neff M (2005). Incidence and outcomes of acute lung injury. N Engl J Med..

[22] Santana-Cabrera L, Lorenzo-Torrent R, Sánchez-Palacios M, Martín Santana JD, Hernández Hernández JR (2014). Influence of age in the duration of the stay and mortality of patients who remain in an Intensive Care Unit for a prolonged time. Rev Clin Esp (Barc).

[23] Muratore S, Hawes L, Farhat J, Reicks P, Gipson J, Beilman G (2016). Riding into the golden years: injury patterns and outcomes of advanced-age motorcycle trauma. Am J Surg.

[24] Cislaghi F, Condemi AM, Corona A (2009). Predictors of prolonged mechanical ventilation in a cohort of 5123 cardiac surgical patients. Eur J Anaesthesiol.

[25] Ross BJ, Barker DE, Russell WL, Burns RP (1996). Prediction of long-term ventilatory support in trauma patients. Am Surg Am Surg.

[26] Agle SC, Kao LS, Moore FA, Gonzalez EA, Vercruysse GA, Todd SR (2006). Early predictors of prolonged mechanical ventilation in major torso trauma patients who require resuscitation. Am J Surg.

[27] Dimopoulou I, Anthi A, Lignos M, Boukouvalas E, Evangelou E, Routsi C (2003). Prediction of prolonged ventilatory support in blunt thoracic trauma patients. Intensive Care Med.

[28] Kung S-C, Lin W-T, Tsai T-C, Lin M-H, Chang C-H, Lai C-C (2017). Epidemiologic characteristics and outcomes of major trauma patients requiring prolonged mechanical ventilation. Medicine (Baltimore)..

[29] Flagel BT, Luchette FA, Reed RL, Esposito TJ, Davis KA, Santaniello JM (2005). Half-a-dozen ribs: the breakpoint for mortality. Surgery.

[30] Bulger EM, Arneson MA, Mock CN, Jurkovich GJ (2000). Rib fractures in the elderly. J Trauma Injury Infect Crit Care..

[31] Holcomb JB, McMullin NR, Kozar RA, Lygas MH, Moore FA (2003). Morbidity from rib fractures increases after age 45. J Am Coll Surg.

[32] Pressley CM, Fry WR, Philp AS, Berry SD, Smith RS (2012). Predicting outcome of patients with chest wall injury. Am J Surg..

[33] Pape HC, Remmers D, Rice J, Ebisch M, Krettek C, Tscherne H (2000). Appraisal of early evaluation of blunt chest trauma: development of a standardized scoring system for initial clinical decision making. J Trauma Injury Infect Crit Care.

